# Ten simple rules for faculty members building just and equitable environments in academic science

**DOI:** 10.1371/journal.pcbi.1013177

**Published:** 2025-07-07

**Authors:** Veronica Martinez Acosta, Carlita Favero, Barbara Lom, Deanne Buffalari, Amy Jo Stavnezer, Leah Chase

**Affiliations:** 1 Department of Biology, University of the Incarnate Word, San Antonio, Texas, United States of America; 2 Biology Department & Neuroscience Program, Ursinus College, Collegeville, Pennsylvania, United States of America; 3 Biology Department & Neuroscience Program, Davidson College, Davidson, North Carolina, United States of America; 4 Department of Neuroscience & Psychology, Westminster College, New Wilmington, Pennsylvania United States of America; 5 Neuroscience Program and Psychology Department, College of Wooster, Wooster, Ohio, United States of America; 6 Biology and Chemistry Departments & Neuroscience Program, Hope College, Holland, Michigan, United States of America; Carnegie Mellon University, UNITED STATES OF AMERICA

## Introduction

**Fig 1:**
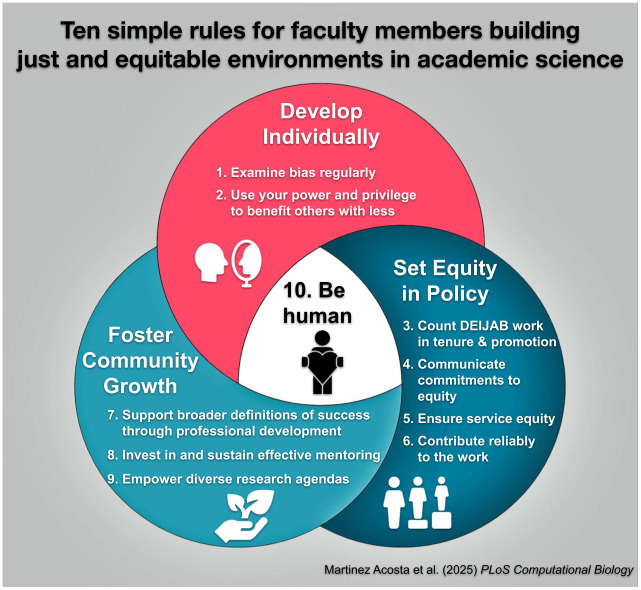
Rules that foster just and equitable academic science environments. We all must take actions to develop as individuals, to ensure policies reinforce equity, and to foster communities of continual growth to build and sustain just and equitable academic STEM environments. It is important to note that the very simple rule of being human (#10) and connecting with colleagues is central and foundational to the nine suggested rules for building equity-minded communities through personal (#1–2), policy (#3–6), and community (#7–9) efforts. Icons from thenounproject.com through an Icon Pro membership.

We recognize that, like us, many academics want to contribute in meaningful ways to improve a sense of belonging in academic culture and may be looking for practical guidance on achievable actions for themselves, their departments, or institutions. We offer these “Ten Simple Rules” (Fig 1) for creating just and equitable environments in academic science as six tenured female STEM faculty members, who have also served in administrative roles at numerous academic institutions and professional organizations within the US and who each bring a variety of unique and intersectional identities, some of which are marginalized and others that are dominant. Together we have engaged in repeated conversations related to non-promotable labor and our individual actions (often rooted in examples from the literature) to improve inclusivity in our classrooms, labs, and institutions. We share these suggestions based on a variety of experiences but also from a place of compassion and engagement in the ongoing work. Though the literature already offers much guidance, we crafted these Ten Simple Rules (TSR) as a reference for those interested in improving the cultures in which they work. We recognize that given our collective experience, the contextual background for these suggestions is primarily focused on American colleges, universities, and academic organizations. Despite this limitation, we believe these ideas are globally applicable, but will likely need to be adjusted to fit the particulars of other environments. In Part 1 (“Develop Individually”, Rules 1–2), we encourage each of us to engage in ongoing implicit bias education so that we can reflect on our own unconscious biases and learn how to use this knowledge to advocate for and uplift our colleagues. In Part 2 (“Set Equity in Policy”, Rules 3–6), we suggest ways that we can each champion policy and procedural changes at our institutions that result in improved work satisfaction and experiences of equity [2]. In Part 3 (“Foster Community Growth”, Rules 7–9), we advocate for professional development, mentorship, and validation of research topics beyond traditional disciplinary norms as critical steps for broadening representation in the academy. Finally, we remind our readers that connecting with others and deploying gratitude in our daily work is a powerful mechanism for positive cultural change (Rule 10). Barriers to success in STEM are numerous—some are under our control because we are responsible for the relational systems within which we work. We share these rules as routes to promote equitable and inclusive opportunities for more scientists to thrive by improving a personal and professional sense of mattering. We also note that climates for supporting academic science can rapidly, unexpectedly, or discouragingly change [3]. Consequently, ensuring access, justice, and support for scientists of all identities to contribute, thrive, and advance scientific knowledge in welcoming spaces is even more important than ever [4].

### Without individual development, STEM and academia will remain unwelcoming for those who are marginalized

As academic scientists and educators, we know diverse groups produce more successful scientific outcomes [5]. Yet, members of the academy do not represent the full diversity of the population [6]. A lack of STEM advanced degree attainment [7], underrepresentation in the STEM workforce [8], diminished promotion to advanced ranks and leadership roles, and lower salaries [9] for members of underrepresented groups all confirm that our scientific community does not allow every individual to contribute to their full potential. Why? Because “the cumulative and compounding effects of an array of racialized societal factors—including the history, culture, ideology, and interactions of institutions and policies that systematically disadvantage people from marginalized groups—create substantial barriers that make it difficult for almost half of the nation’s population to join and prosper in the STEM workforce” [7]. We use the term marginalized, in line with the published literature, to represent individuals in STEM who are negatively affected by historical and current forces that reduce their power, opportunities, and/or significance. These systemic issues lead to an academy wherein white, cis-males are more often highly represented and promoted [7]. A wealth of information describes how to achieve more diverse and equitable scientific environments [7,10–14]. Many individuals working in academic science certainly recognize that a community of inclusion where all members feel a sense of belonging is the right thing to do [13,15]. Yet follow-through on such initiatives can be minimal [16]. Thus, we clearly have work to do to build a thriving and diverse scientific enterprise. Because the culture of academia posits that successes are based on meritocracy systems with objective measures [17], many within academia believe they are immune from bias and do not contribute to the inequities within the scientific community. Yet data demonstrate that inequities persist and are likely driven in part by personal biases; numerous studies have shown that faculty members select candidates with white male-sounding names over female-, Latinx- or Black-sounding names for academic positions [18–20]. Inequities in tenure, promotion, salaries, resource, publication, and citations have not been remedied by broadened access to academia [21,22]. Given that individuals within the scientific community make decisions about who gets hired, tenured, funded, and cited, inequities are likely to continue unless those individuals seek change. Thus, academics must reduce their own implicit biases with the goal of creating a diverse, inclusive, justice-minded academy.

### Structures and systems must have equitable policies to improve inclusion

Although addressing personal bias is an important first target, academic cultures need clear, equitable, and transparent policies that delineate how success can be achieved. Current professional development or on-boarding methods fail to recognize the increasing diversity of current and incoming colleagues and students and the need for support in implementing thorough equity-minded practices at all levels. Consequently, faculty members may hesitate to engage in evidence-based educational and professional practices that support diverse students and colleagues. Others do not have opportunities to do so. Existing organizational structures may also fail to acknowledge and incentivize multiple paths to success. In systems where job expectations are often unclear [23] and communicated via informal social networks [24], individuals who do not occupy positions of power can be disadvantaged [25,26]. For example, a lack of clarity regarding what counts as research versus service can make tenure and review processes opaque [27]. Individuals who extend to work outside of their primary research training may jeopardize their scholarly output, delay their research trajectory, compromise collaboration opportunities, and/or negatively affect their mental health [10,28]. It is unsurprising, then, that a lack of clear communication is associated with inequitable career outcomes [24,29], whereby those underrepresented in science are inadequately supported to achieve success, or their successes are deemed insufficient. Therefore, even with significant policy reform to improve access, inclusion, and varied paths to success, all individuals in academia must be well-versed and well-supported in their paths to success. Further, colleagues making decisions regarding the success of others should do so in a way that aligns with policies that promote equity and justice. Only then can personal growth, improved policies and procedures, and developmental support come together to achieve the goals of inclusion and equity set out years ago.

### Successfully navigating and finding community within STEM and academic environments is particularly challenging for those with marginalized identities

The Academic Council for Educational Accountability and American Association of University Professors long ago called for greater clarity and transparency in academia to support improved outcomes and equity, and that transformation is far from realized [30]. Studies indicate that adequate and positively assessed mentoring programs have yet to be realized for between half and two-thirds of junior faculty members [31–33]. In addition, faculty members at private universities have more opportunities for formal mentoring [34]. Inequities in tenure, promotion, publication, and funding rates are not a result of lack of inherent ability or interest, but instead due to the meritocracy framework of policies and procedures (e.g., tenure and promotion guidelines) that are designed to disadvantage those from marginalized groups [7]. Publication rates among Black and Hispanic scientists do not align with their representation in science [35] and in papers with similar content, white and Asian-Pacific Islander authors are cited more often than non-white authors, a trend that has persisted for decades [36]. Further, disciplinary norms often value inquiry-based research [27], despite the fact that some marginalized faculty members are more likely to work in areas of engaged scholarship or with marginalized populations [27,37–39]. This research often aligns with institutional initiatives toward diversity, equity, inclusion, justice, access, and belonging (DEIJAB), which is often considered service rather than scholarship and therefore devalued. Revisions of the “rules” through which success in academic research are measured are long overdue. Improved policies and procedures are also needed to make service expectations more valued and more equitable. Women and faculty members with marginalized identities spend more time on service, committees, student mentorship, DEIJAB efforts, and departmental “housekeeping” [5,6,20–26] and have fewer opportunities for adequate mentorship [32]. An identity or culture tax expects female and marginalized individuals to take on more mentorship and/or advocacy because their identities are sought out by committees seeking diverse representation, colleagues, and students from similar backgrounds who see few other potential mentors with shared identities [5,6,19,20,22,23,26]. Given that time is finite, efforts spent on these non-promotable activities reduce time to engage with research and writing, reduce productivity, increase burnout, decrease retention, and extend the timeline to tenure and promotion [9,39,42–47]. Thus, it is critically important that academic scientists and educators use best practices to establish equity-minded, transparent policies and procedures that recognize each individual’s holistic contributions to our institutions.

### Part 1 Develop Individually

1 Examine bias regularly

Though no single action will result in inclusion in academic science, examining biases is a necessary first step. Without knowing our unconscious biases and culturally shaped beliefs [48], we risk contributing to the problem(s). Many of us believe we are unbiased because our explicit behaviors demonstrate equity, yet at times our implicit responses or thinking can be contradictory [49–51]. Effective implicit bias training programs provide individuals tools for changing their behaviors by helping individuals better understand others’ experiences and increasing motivations to change (See Box 1; [52,53]). Engaging in implicit bias training that is primarily focused on raising awareness is insufficient [54,55] because simply gaining knowledge about unconscious bias does not necessarily translate into an understanding of the concrete steps that can be taken to result in meaningful actions and policy changes [56]. Therefore, we should seek out implicit bias training such as the “gender-bias-habit changing intervention” workshop designed by Devine and colleagues [50]. When compared to non-trained colleagues, STEM faculty members who participated reported increases in self-action to promote gender equity, greater perceptions of fit, feeling valued in their research, and felt more comfortable raising personal and professional conflicts [57]. In addition, departments participating in the training increased the proportion of women hired relative to departments that did not undergo intervention training [50].

Although professional development opportunities may not be available, core components of this training can be intentionally constructed by individuals or groups. A simple first step is to take a free implicit association test (IAT; implicit.harvard.edu) to raise awareness of our implicit biases [51,58]. Other examples of accessible starting points include awareness training on: anti-racism [59, 60, 61], allyship [59], and privilege [60]. Being intentional in getting to know our colleagues with backgrounds and experiences different from our own is another important personal action. The more we are able to individuate and move away from stereotypes, the less implicit bias we tend to perpetuate [52,61,62]. We must maintain growth mindsets [63,64] in this work, as the work to face our own biases can be emotionally challenging, though ultimately critical to personal growth and change. More powerfully, when institutions require and provide bias training then all individuals in the academic environment, including those who might not otherwise engage in examinations of bias, can develop shared understandings, language, and tools.

Box 1 **Key elements of prejudice/gender habit-breaking interventions [52,57]**

1. Complete Implicit Association Test (IAT) (implicit.harvard.edu)
2. Review evidence of continuing racial/gender bias in STEM and the role of implicit bias
3. Learn bias constructs (expectancy bias, prescriptive gender norms, role congruity/incongruity, stereotype priming, reconstructing credentials, and stereotype threat)
4. Learn to use evidence-based strategies that effectively counteract unintentional bias
5. Write statements of commitment to address bias in personal and professional life using the strategies learned

2 Use your power and privilege to benefit others with less

Everyone has some degree of power and privilege [1,65]. These advantages make us less likely to be aware of inequities experienced by our marginalized colleagues because we have been socially buffered. We need to invest time and cognitive bandwidth in ongoing introspection and to process the effects of oppression due to marginalized identity status so that we can be open and value alternate perspectives. To be supportive colleagues, we need to recognize the privileges we hold due to our identities, experiences, and positions. We also must remain attuned to dynamics of empowerment and disempowerment in a variety of contexts [45]. Upon recognizing these privileges and how they impact the environments in which we and our colleagues work, we must consider how to use these unearned assets to dismantle similar systems and share power.

We can use our position(s) to assist and advocate for colleagues who do not hold power and privilege in the areas we do, so they may navigate often tricky academic environments both successfully and sanely [66–69]. We advance equity by sharing and giving away our privilege [70–72], pointing out practices that do not promote equity, and suggesting opportunities to reduce marginalizing conditions [73]. We can advocate within our spheres of influence, whether one-on-one interactions or from formal leadership platforms. One goal in creating collegiality is to move from a place of competition or comparison among colleagues to a culture that provides and expects support. Actions may include (1) encouraging colleagues to consider opportunities given the evidence that those in marginalized groups often discount their own readiness or appropriateness [4,74], (2) speaking out when we observe policies with unstated, unwritten, and often esoteric expectations, and (3) being sure to give credit when and where it is due rather than allowing the actions or ideas of some to be undervalued. The collective work of individuals in research labs, classrooms, department meetings, journal clubs, and lunchrooms questioning problematic practices that fail to acknowledge power, privilege, and status results in equity becoming a priority and value of our institutions. This essential work within our reachable spheres of control, influence, and concern [75] is how we begin to transform the complex patterns of unstated academic expectations into clearly articulated policies that disrupt the practices that perpetuate exclusion and marginalization in academia.

### Part 2 Set Equity in Policy

1. Count diversity, equity, inclusion, justice, access, and belonging work toward tenure and promotion

Faculty members with marginalized identities are often heavily engaged in DEIJAB work, which can be high-intensity and long-lasting. This situation may contribute to disproportionate service burdens. A variety of reasons cause DEIJAB work to fall consistently on the shoulders of faculty members with marginalized identities including institutional cultures and norms, a greater likelihood to say yes to maintain their position in a department and/or to be seen as a team player, to defy racialized or gendered stereotypes, and because students and administrators seek out their specific identity [38,76–80]. We can circumvent the culture tax, which expects marginalized individuals to take on more mentorship and/or advocacy because of their identity, by thinking carefully before we ask for assistance, creating and checking a dashboard designed to distribute service burden (see Rule 5), considering others who can also champion a particular cause, and allowing colleagues ample time to consider a request before responding [9,10,39,40,43,47,79,80].

More importantly, it is critical that we advocate in our departments and institutions for DEIJAB work to count toward tenure and promotion. This work is critical to institutional missions [41,43,81,82]. For some faculty members with marginalized identities, DEIJAB efforts align with scholarship, personal values, and/or advocacy work [83,84]. Advising, mentoring, and supporting marginalized student and faculty populations as well as departmental or institutional equity efforts need not fall only on the shoulders of marginalized faculty members if we all present a concerted effort toward allyship [85]. By designating DEIJAB work as valuable, and validating it as a marker of professional success, not only will we avoid “penalizing” those who currently engage in this important work, we will incentivize others to get involved.

4 Communicate commitments to equity

Effective communication is critical as we work towards a more equitable community. Communication is a cornerstone of effective scientific collaboration, leadership, and mentorship [86–89]. Yet our academic organizational structures may suffer from “hollowed collegiality;” fragmented communication can isolate faculty members and limit cooperation and effectiveness [90]. “Hidden rules” allow gate-keepers to limit access to success, upper ranks and/or leadership positions [30,84,91]. Such “strategic ambiguity” perpetuates inequality [24,32,84] as individuals with marginalized identities are most affected, creating reduced morale, lower job dissatisfaction, and departure from scientific positions [2,29,32].

Therefore, as members of the scientific community invested in equity and justice, we must improve our communication. We communicate informally with our own voices, but also formally via written rules, guidelines, and policies. We should all engage in rigorous training in communication [32,89,92] to become effective advocates, allies, and sponsors [66,67,92–94], particularly those of us in leadership roles. But we must also work with our colleagues and leaders to revise the policies and procedures that communicate to scientists how they can achieve success, and use our voices to insist to those same colleagues and leaders that those rules are transparent, widely accessible, and followed. These actions can happen at the level of a department or school, but are also important in institution-wide policy as well as other entities that impact success- editorial boards, disciplinary organizations, and beyond. We must ask for and practice inclusive communication that builds community [71] at all levels: respectful personal communication, explicit statements of laboratory values, equitable guidelines that govern participation and success. Varied mechanisms to solicit and listen to feedback from all members are vital to productive work in academia/science [95,96]. Such efforts can seek, support, and amplify the voices of all individuals.

As we work to revise policy, we should do so with diverse groups, and break away from rules designed to advantage the historical majority [7,72] to allow diverse paths to success. We must ensure policies are broadly and regularly communicated to both those navigating the system and those in positions of power who judge success. Perhaps most importantly, we can use our voices to insist that these improved policies are put into practice; we must reject academia’s long-standing culture that limits what our colleagues can do in favor of a more positive, equitable, and transparent professional environment moving forward.

5 Ensure service equity

Faculty members with marginalized identities spend more time on service-related activities, removing time from tasks more highly valued by the academic reward structure [47,79,82]. Articles and books that encourage faculty members to “just say no” [97–99] are well-intentioned with excellent advice to individuals who are navigating a sea of requests, yet they can dismiss the fact that the work must get done [2]. Recruiting, mentoring, and retaining students at all levels is a key to the success of higher education [83,100]. Departments will not be successful if no one chairs the committees, meets with prospective students and faculty candidates, records minutes, advises and mentors students, plans celebrations, or takes initiative on inclusive teaching practices. These tasks require time, a finite entity [47,79,101], and deserve to be legitimized and considered promotable tasks.

To address workload disparities, it is critical that we, along with our academic units, consider the mechanisms by which service and teaching assignments are allocated and understand how and why inequities develop. We must pursue a comprehensive approach to changing the “choice architecture” [2,10] within a department. We can work with our colleagues to establish a “dashboard” system [2,10,37,38]. An empirical study demonstrates that dashboards made public across the department are effective in establishing departmental conditions that lead to equitable workloads [38]. The key components that improve departmental workload equity include: (1) transparency, (2) clarity, (3) credit, (4) norms, (5) context, and (6) accountability [38]. O’Meara and colleagues have carefully detailed the process and workflow that departments can follow to establish dashboards (See Box 2; [10]). Although this process can lead to some uncomfortable discussions as patterns of inequity may be revealed, the improvement in faculty job satisfaction and perception of equity in service loads can result in increased faculty retention and an improved departmental culture. Bringing all service activities to light also provides increased opportunity to recognize essential contributions by colleagues in tenure and promotion processes.

Box 2 **Steps in creating faculty dashboards and equitable service loads [10]**

1. Review example faculty workload dashboards
2. Complete a faculty service audit
3. Develop faculty expectation guidelines
4. Establish compensation system for key roles
5. Establish a credit system for other teaching/service activities
6. Create a plan for service and teaching rotations
7. Establish a differential workload policy that recognizes the strengths of individual faculty members

6 Contribute reliably to the work

Though beneficial, a dashboard will not address issues of social loafing, wherein a department member does not pull their weight on specific assignments or roles [10]. Although multiple means of assessing teaching and scholarship are available, few benchmarks are in place to assess quality of academic service work. Some department members regard service assignments as distractions from their scholarship and thus rely on others to complete the tasks or do poor jobs in those roles to minimize the chances of subsequent service expectations. This inequity often leads to group dysfunction and dissatisfaction, diminishing the effectiveness of the team in completing the task [102,103].

Social loafing has been studied by sociologists who suggest workload inequities develop when committee size and leadership roles are not carefully considered [104]. Therefore, it is critical to advocate for the establishment of small committees that are more effective than large committees in increasing individual accountability [105]. It is important to make each individual’s work visible, able to be evaluated clearly, to establish normative standards and expectations of committee members at the outset of [106] and be diligent in not allowing others to carry your load or the loads of others. We must resist the urge to pick up the slack for a colleague who does not participate or to allow other committee members to compensate for those not putting in time and effort to balance the system. This last bit of advice can be particularly hard for some academics to follow, particularly those in more vulnerable positions. Therefore seeking the support of allies and those in your support network can be a helpful strategy when you find yourself tempted to fill in for others. Again, faculty members in positions of power can use their voices to insist on policy and procedures that promote equitable contributions to the work. All such efforts will lead to more equal distribution of the service tasks of a department and institution over time so that faculty members with marginalized identities do not end up shouldering a greater share of service loads.

### Part 3 Foster Community Growth

7 Support broader definitions of scientific success through professional development

The concept of success in academic science is typically personified as attainment of a tenure-track faculty position at a well-respected doctoral granting institution where degree of accomplishment over career span is measured in rank status, high-impact publications, and a small fortune in federal grants [107,108]. Citation counts, often used as an “objective” metric of success, not only perpetuate gender and racial biases, but actively harm the career advancement of marginalized groups [108]. Furthermore, citation counts exclude important scientific contributions such as early-career training of future scientists, scientific communications in the public realm, policy-making, and Indigenous knowledge systems [108,109]. Many academics feel constrained by this definition of success and prohibited from engaging work that is fulfilling to them personally and professionally [107,108]. Further, this definition of success presumes a personal failing when individuals are not able to attain it rather than examining the systems and structures at play [110]. Expanding our definition of success and providing appropriate support will enable all members of the professoriate to thrive. We cannot expect change without investing in systems and structures that incentivize, normalize, recognize, and expect ongoing professional development that is particularly aware of the barriers faced by individuals who belong to marginalized groups to advance at all levels. Training of all professionals in culturally responsive pedagogy and professional practices, including how to become better sponsors for those who are marginalized [111–113] is an important start. Only when we design and embrace professional development as a supported, required, and essential practice within the academy can we progress to a place where individuals and institutions are equipped with the resources needed to promote success.

Faculty development centers, programs, and professionals are well-positioned to educate individuals in the many roles they assume as academic scientists and work together in units such as departments, research groups, classrooms, and committees. When possible, we should lobby to strengthen these positions, offices, or groups to ensure that advancement of equity and diversity is an institutional priority that is embedded into our daily practices [13,14,85]. Similarly, scientific organizations are poised to offer support for a broad sector of academic science through their membership which often includes individuals at different stages of career and different institution types (see the following for actions scientific societies are taking to support their diverse membership [114–118]). Improved support for the diverse scholars that enter science can help them overcome barriers to success. Professional development opportunities that focus on the positive outcomes associated with a broadened definition of success can help those in evaluative positions better understand that a difference is not a failing. In teaching, for example, we can value using evidence-based best practices in the classroom (e.g., alternative grading strategies, service learning) and do away with outdated, inherently biased metrics such as student evaluations [24]. In service, we can value mentorship that is marked by increased sense of belonging, career satisfaction, recruitment and retention among members of marginalized groups ([108]; see more in Rule 8). In scholarship, we can support the work required to establish research programs (e.g., attending conferences that may not be evidenced by research presentations, participating in grant and article writing workshops). We can also give equivalent weight to scholarly outputs that are not hypothesis-driven research (see more in Rule 9). If we can cultivate a scientific community of support (rather than competition), we may be more accepting of differences, welcome opportunities to learn from our colleagues, and find that our own practices improve as a result. Not only will our colleagues feel valued and supported by these actions, but an expanded definition of success will result, promoting diversity and continued innovation in STEM. We posit that professional development initiatives should be a supported, required, regular, and essential part of faculty work.

8 Invest in and sustain effective mentoring

Scientists who belong to marginalized groups are more likely to experience mental health challenges (e.g., depression, anxiety) so it is imperative that mentoring relationships challenge toxic environments and promote safety and well-being [108]. As Zambrana and colleagues (2015) implore us to recognize, “Effective mentorship of URM [underrepresented minority] faculty is everyone’s responsibility if we are to transform the academy into a space where all faculty can thrive” ([32], p. 69). We would extend this statement and ask that we recognize the importance of mentorship to all in the academy. Despite widely heralded training for inclusive research mentoring for students (see: https://cimerproject.org/), many scientists tend to invest less in developing this important skill as we move into faculty, leadership, and administrator roles. The majority of formal mentoring programs are still didactic in nature, which can lead to limited perspective-taking [31] and does not align with recent initiatives led by the National Center for Faculty Development and Diversity which supports mentoring networks based on nine core categories of support ([119]; See Box 3). In addition the traditional hierarchical mentoring model, with more senior colleagues serving as mentors, does not align with the increasing number of successful, formalized peer mentorship programs [120,121]. Developing a community or village of support is especially important to those who are marginalized and rarely find themselves in groups with people who have shared experiences [32,122,123].

Box 3 **Critical areas of support for faculty development [119]**

1. **Substantive Feedback**
   people who can offer constructive criticism on all aspects of your job
2. **Sponsorship**
   people who have power, know you, and can advocate on your behalf to influence your trajectory positively
3. **Access to Opportunities**
   people in professional networks who can give you the inside scoop (e.g., grant funding options, course releases)
4. **Accountability**
   people who check in on your progress on personal and professional goals that have little to no external accountability
5. **Professional Development**
   people and organizations that can offer guidance and training as you embark on endeavors that are new to you
6. **Emotional Support**
   people, perhaps outside your institution, where you can speak truthfully and freely in challenging times
7. **Role Models**
   people who are doing life (academic and otherwise) in ways that you aspire to do
8. **Intellectual Community**
   people who can comment on your scholarship at every stage of the progress toward completion
9. **Safe Space**
   places where you can relax, bring your whole self, and speak in an unfiltered way without being judged

The good news is that a review of the literature found no “best” practice for mentorship programs [124], indicating that institutions can individualize their efforts, provided they include some basic parameters *(see* [123] *for case studies of three programs)*. Mentorship programs have been successfully initiated across institutional levels, but support from higher level administration influences success [34]. Support can take the forms of stipends, course-release, competitive grants, or formalized recognition in annual reviews [34]. Additionally, mentoring programs and relationships should be monitored and assessed to optimize their outcomes [108]. Successful mentoring communities welcome input from the mentees and include information on: navigating and deciphering unwritten rules of the university system and culture, knowledge about promotion and tenure expectations and career advancement, introductions to scholarly networks and advocacy for opportunities, and constructive feedback. This work can be shared by creating small cohorts with several mentees and a few senior mentors or by formalizing peer support groups which help to increase collegiality, decrease professional isolation, increase areas of expertise, destigmatize failure, and allow research and teaching discussions in collaborative environments that minimize fear of judgment [31,120,125]. A key to all of these efforts is the development of trusting relationships and programmatic acceptance of the importance of mentorship, which speaks to several other rules in this paper – academic scientists must be open to experience, equity-minded, and supportive of differences if we are to increase the diversity of our science and colleagues. Most faculty members who reported they had considered leaving their positions had inadequate mentoring [31], indicating that we must mentor well because it increases retention and promotion [108,121]. Finally, each of us can demonstrate support by intentionally providing safe spaces to have difficult conversations and by connecting colleagues to others, inside or outside of our institution, who have traversed similar professional pathways [111,122].

9 Empower diverse research agendas

Due to societal and culturally based implicit biases, academics in marginalized groups experience epistemic exclusion (a.k.a. academic hazing) that invalidates their research and scholarship [27]. Disciplinary norms often prefer inquiry-based research within the mainstream dogma [27], areas where researchers from marginalized groups may be less likely to engage [10,27,37,39]. As colleagues and evaluators, we need to value diverse ways of knowing, varied qualitative methods, and engaging with community partners to shift outdated perceptions of disciplinary norms. We can individually learn about our colleague’s fields, help to contextualize their work, and gather evidence of their impact in nontraditional ways [10,96,113]. We can advocate to move away from journal impact factors and high-profile journals as criteria for success, recognizing that journals traditionally considered prestigious are less likely to publish outside central disciplinary dogma, on engaged pedagogy, and/or with populations of non-white participants [27].

As a peer reviewer or editor, we can shift disciplinary norms and prioritize diversity in our science and membership. Editorial boards are commonly staffed with >75% males [126] with Black and Hispanic scientists being grossly underrepresented (0.1% of editors are Black [36]). Black majority authorship manuscripts experience significantly longer delays to acceptance [36] and manuscripts discussing marginalized populations are often subjected to white hegemonic reviews that invalidate the scholar’s knowledge and require extensive explanatory revisions to satisfy a white audience [127]. Recent evidence suggests the lower number of publications by women is not directly related to productivity, but inequity, implicit bias, and disciplinary norms that can result in lack of authorship [128,129]. These challenges can lead to fewer grants, delayed career advancement, smaller or no raises, and decreased retention [36,129]. We can work to reverse these trends by being aware of our disciplinary biases and familiarize ourselves with the paradigms and limitations of the field when reviewing applications, portfolios, manuscripts, and grant proposals. We can encourage journals and colleagues to use CRediT (Contributor Role Taxonym) for determining authorship to recognize associated scholars more equitably [130]. We can actively discuss the importance of representing all of scholarship, not just the dominant methods, content, or techniques to prevent gatekeeping [18,107]. We can approach reviewing as a collaborative rather than confrontational process by encouraging authors to take on the challenge of revision by lifting up the strengths in their work [131], again reinforcing a collaborative and supportive scientific community. We can also advocate for anonymous review by editors and panels for manuscripts and grant proposals to alleviate the historical bias against underrepresented groups in these fora [132,133].

10 Be human

Despite the increasingly collaborative nature of STEM and the physical proximity of the campus environment, individuals can easily feel isolated in academic science. At least 40% of academics indicate that isolation at work influences their mental health [134]. The pressure to keep our ‘head down’, reduce distractions, stay busy, favor individual scholarship over collaborations, and pursue funding and promotion in constant comparison with others can exacerbate isolation [135,136]. This danger is especially pronounced in individuals who are marginalized. Working in a system that emphasizes competition and productivity reduces opportunities to get to know colleagues as unique and multi-dimensional individuals and can breed misconceptions about colleagues that affect important decisions regarding professional trajectories.

Science or any creative pursuit does not, should not, occur in silos. We need to invest in relationships [137] and make time to check in often with ourselves and others. We can seek and offer help, collectively acknowledge the joy in the work we do to understand the natural world, uplift our colleagues on good days and bad [138], and celebrate successes of all kinds (not just professional accomplishments). To overcome the public health concerns of loneliness [139] we must intentionally create connections [140,141]. We can keep eating lunch at our desks or enjoy lunch (one day each week or month) with colleagues. We can grab a coffee with a colleague and then visit for a bit [142]. We can use the first five minutes of meetings for sharing recent moments of growth or success. When we know people we are better positioned to mention opportunities, nominate them for awards, provide a sounding board, and support each other [135].

Practicing gratitude is a simple way to help our colleagues feel seen and let them know that we care about them and the work they do [135]. Gratitude is also important for our own personal well-being and success [143]. Importantly, being grateful does not deny or ignore the negative aspects of academic life, but those who are grateful are more empathetic, giving, and less likely to compare what they have and do with others [143]. Practicing gratitude by keeping gratitude journals, gratitude lists, or self-guided exercises leads to: (1) improvements in feelings of health (physical and mental), (2) increased progress on reaching important goals (academic, interpersonal and health), (3) higher levels of alertness, enthusiasm, determination, and energy, and (4) increased likeliness to offer emotional support to others [143].

To expand our gratitude, we need to remember to say thank you. We need to say it early and often. We need to say it to everyone who impacted us positively, regardless of status, rank, title, or level of achievement. Appreciation is a powerful action that can profoundly improve us, our academic environments, our colleagues, and our communities of science. We were all humans first before we learned to be scientists; extending kindness, showing care, and investing in stronger relationships are critically important actions well within our reach [137].

## Conclusions

The benefits of building and sustaining more diverse scientific academic communities are numerous. Scientists are asking increasingly complex and urgent questions; diverse teams have strong potential to make important advances/insights [5,144] because diverse researchers bring disparate perspectives and experiences [145,146]. Scientists with varied backgrounds are often passionate about addressing questions that directly relate to their life experiences, and those with non-traditional expertise address questions with unique synergistic approaches. Yet, the diversity of the scientific community does not reflect the diversity of the general population (most US children are not white and half are women [147]). As scientists, we each need to prioritize the well-being, effectiveness, and diversification of our scientific communities. We can improve opportunities for everyone to thrive by making sure that service and work loads are equitable, knowledge and expectations are disseminated clearly, and by simply letting our colleagues know that they matter. Our recommendations suggest steps that everyone in a professional community can take to support individuals from marginalized groups. It is also important to note that even in times when DEIJAB training is challenged, there is still much value in the offering of support that reflects the experiences, strengths, needs and challenges of all individuals within the scientific enterprise [148–152]. Indeed, the work continues, despite the barriers that arise, because we need all individuals to thrive.

Developing these TSR allowed us to engage with a diverse and meaningful literature, and we hope our readers might also find inspiration in the good work these many authors are doing to advance just and equitable STEM environments. We recognize that academic scientists are often overburdened with numerous responsibilities, and at first glance, this list of rules may seem overwhelming and challenging to enact. Yet it is our sincere hope that as a reader you are inspired to take actions within your professional communities as you are able. The most important message is—do something to be part of the solution by expanding your capacity, supporting your colleagues, and improving practices. Collectively, our contributions will not only broaden the diversity of our scientific communities, but will allow science to make important, otherwise impossible, progress and insights in exciting new ways.
